# Gly322Asp and Asn127Ser single nucleotide polymorphisms (SNPs) of *hMSH2* mismatch repair gene and the risk of triple-negative breast cancer in Polish women

**DOI:** 10.1007/s10689-014-9746-z

**Published:** 2014-08-19

**Authors:** Beata Smolarz, Marianna Makowska, Dariusz Samulak, Magdalena M. Michalska, Hanna Romanowicz

**Affiliations:** 1 Laboratory of Molecular Genetics, Department of Pathology, Institute of Polish Mother’s Memorial Hospital, Rzgowska 281/289, 93-338 Lodz, Poland; 2 Regional Hospital in Lodz, Lodz, Poland; 3 Department of Obstetrics and Gynaecology, Regional Hospital in Kalisz, Kalisz, Poland; 4 Cathedral of Mother’s and Child’s Health, Poznan University of Medical Sciences, Poznan, Poland; 5 University of Computer Sciences and Skills, Lodz, Poland

**Keywords:** hMSH2, MMR, Breast cancer, Gene polymorphism

## Abstract

Triple-negative breast cancer (TNBC) is characterised by worse clinical outcome and poor prognosis. The alterations in the oncogenes and tumor suppressor genes as well as microsatellite instability (MSI) have been associated with breast cancer development. It is knowledge that the most common mechanism inducing MSI in many cancer is genomic rearrangements found in the *hMSH2* (human MutS homolog 2) gene. In this report we genotyped two polymorphisms of *hMSH2* DNA repair gene in 70 TNBC patients and 70 age-matched cancer-free women using RFLP–PCR. The following polymorphisms were studied: an A/G transition at 127 positions producing an Asn/Ser substitution at codon 127 (the Asn127Ser polymorphism, rs17217772) and a G/A transition at 1032 position resulting in a Gly/Asp change at codon 322 (the Gly322Asp polymorphism, rs4987188). We found an association between the *hMSH2* Asp/Asp and Gly/Asp genotypes and TNBC occurence. Variant Asp allele of *hMSH2* decreased cancer risk [odds ratio (OR) 0.11; 95 % confidence interval (CI) 0.05–0.21]. The risk of TNBC in the carriers of the Gly322Gly–Asn127Ser combined genotype was increased (OR 3.71; 95 % CI 1.36–10.10). However the risk of TNBC was not alter by polymorphism Asn127Ser of the *hMSH2* gene. The Gly322Asp polymorphism of the *hMSH2* gene may be linked with TNBC occurrence in Polish women.

## Introduction

Over the past decade, the landscape of breast cancer has changed. Various genetic and environmental factors have been established as causes of breast cancer, which is a genetically heterogeneous disease [1–3].

Modern molecular genetics methods such as microarray techniques have divided breast cancer into several intrinsic subtypes: (1) luminal A, (2) luminal B, (3) human epidermal growth factor receptor-2 (HER-2)-enriched and (4) the “basal-like” subtype. Both luminal A and B are clinically characterized by expression of hormone receptor-related genes, whereas both HER-2-enriched and the “basal-like” subtypes are less likely to express either estrogen receptor (ER) or progesterone receptor (PgR). Moreover, the “basal-like” subtype, one of the most clinically aggressive of the subtypes, is more commonly negative for all 3 markers-ER, PgR, and HER2-hence the “triple-negative” phenotypic classification [4, 5].

Triple-negative breast cancer (TNBC) and basal-like breast cancer are not the same. Some TNBCs have basal-like genetic patterns, but not all do. While most basal-like breast cancers are triple-negative, a small but significant number may be hormone receptor or HER-2 positive. Basal type cancers are frequently defined by cytokeratin 5/6 and EGFRs staining.

Of the estimated 1,000,000 women of breast cancer diagnosed worldwide in 2008, it is estimated that 172,695 will harbor the triple-negative phenotype.

The alterations in the oncogenes and tumor suppressor genes as well as microsatellite instability (MSI) have been associated with breast cancer development [6–11]. It is knowledge that microsatellites, are particularly prone to replication errors associated with the type of genomic instability that results from the loss of mismatch repair (MMR) function or alterations in MMR genes [12–14].

Mismatch repair deletes mispaired bases resulting from replication errors, recombination between imperfectly matched sequences and deamination of 5-methyl-cytosine. The main MMR pathway is initiated by the recognition of a mismatch by the heterodimer consisting of the MSH2 and MSH6 proteins (also called MutSα). MutSα is responsible for the recognition of base mismatches and insertion/deletion (IDLs) in mono- to tetranucleotide repeats. This complex, MutSα, is able to recognize most base–base mismatches and short IDLs [15].

MMR is a highly conserved repair pathway that functions in improving replication fidelity by correcting replication-associated base–base and insertion/deletion mispairs. The MMR mechanisms play an important role in repair of oxidative damage by mechanisms that are not well understood [16]. MMR is essential for maintenance of genome stability [17–20].

High-frequency MSI is detected more frequently in bilateral but not in unilateral breast cancers [21, 22]. Losses of heterozygosity and/or MSI were detected in 83 % of the skin samples from breast cancer patients, which suggest a potential role of MMR in breast cancer susceptibility [22].

Indeed, recently several studies have shown that the most common mechanism inducing MSI in many cancer is the loss of the hMSH2 protein or genomic rearrangements found in the *hMSH2* (human MutS homolog 2) gene [23–26].

There is also some reports associated mutations in MMR proteins genes (predominantly in *hMSH2*) with the initiation and progression of breast cancer [27–31].

Because a little is known on association of *hMSH2* genetic variants and triple-negative breast carcinoma, we studied whether two polymorphisms of this gene: an A/G transition at 447 position producing an Asn/Ser substitution at codon 127 (the Asn127Ser polymorphism) and a G/A transition at 1032 position resulting in a Gly/Asp change at codon 322 (the Gly322Asp polymorphism) may be linked with TNBC risk in Polish women.

## Materials and methods

### Patients

In the present study, paraffin embedded tumor tissue were obtained from 70 women with triple-negative breast carcinoma, treated at the Department of Oncology, Institute of Polish Mother’s Memorial Hospital, Lodz, Poland between 2000 and 2013. The age of the patients ranged in from 36 to 68 years (the mean age 46.2 ± 10.12). Table 1 shows clinical characteristics of patients. The median follow-up of patients still alive at the time of analysis was 38 months (range 2–70 months). DFS (the disease-free survival) was defined as the time elapsed between excision of the primary tumor and the manifestation of recurrent breast cancer or metastasis. The median DFS was 33.5 months (range 7–65 months). Overall survival (the OS) was defined as time between excision of the primary tumor and death because of cancer. The median OS was 27.3 months (range 2–70 months). The average tumor size was 20 mm (the range 17–32 mm). All the tumors were graded by a method, based on the criteria of Scarf–Bloom–Richardson. This system is the most common type of cancer grade classification used today. In this system, there are three factors that the pathologists take into consideration: the frequency of cell mitosis (rate of cell division), tubule formation (percentage of cancer composed of tubular structures), and nuclear pleomorphism (change in cell size and uniformity). Each of these features is scored from 1 to 3, and then each score is added to give a final total score ranging from 3 to 9. The final total score is used to determine the grade in the following way: Grade 1 tumors have a score of 3–5 (well-differentiated), Grade 2 tumors have a score of 6–7 (moderately-differentiated), Grade 3 tumors have a score of 8–9 (poorly-differentiated).

**Table 1:** Characteristics of breast cancer patients (n = 70) and controls (n = 70) with questionnaire data

Characteristics	Number of patients (%)	Number of controls (%)
*Age (years)*		
<45	27 (39)	24 (34)
45–54	15 (21)	18 (26)
55–64	18 (26)	16 (23)
>64	10 (14)	12 (17)
*Family history of breast cancer* ^*a*^		
Yes	25 (36)	22 (31)
No	45 (64)	48 (69)
*Menarche (years)*		
10	5 (7)	3 (4)
11	18 (26)	21 (30)
12	19 (27)	16 (23)
13	13 (19)	15 (22)
14	10 (14)	12 (17)
≥15	5 (7)	3 (4)
*Parity*		
Nulliparous	25 (36)	20 (29)
1	15 (21)	20 (29)
2	16 (23)	12 (17)
3	9 (13)	13 (18)
≥4	5 (7)	5 (7)
*Menopause status*		
Premenopausal	30 (43)	35 (50)
Postmenopausal	40 (57)	35 (50)
*Use of menopausal hormones*		
Never	37 (53)	30 (43)
Estrogen	33 (47)	40 (57)

^a^Family history defined as self-reporting of at least one first-degree relative with known breast cancer

The distributions of clinical characteristics of the patients are shown in Tables 1 and 2, respectively. Control tissue samples were obtained from patients who had undergone biopsy for benign lesions. Control group consisted of age, sex and ethnicity-matched 70 cancer-free tissue donors (Table 1). All patients and healthy subjects were Caucasian. We enrolled only women born and living in central Poland (Łódź region). The study was approved by the Local Ethic Committee of the Institute of Polish Mother’s Memorial Hospital, Lodz, Poland and each patient gave a written consent.

**Table 2:** The characteristic of TNBC patients^a^

Characteristic	Patients N (%)
*Bloom*–*Richardson grading (ductal carcinoma only)*	
1	20 (29)
2	45 (64)
3	5 (7)
*Tumor size grade*	
T1	8 (11)
T2	40 (57)
T3	18 (26)
T4	4 (6)
*Lymph node status*	
N0	32 (46)
N1	12 (17)
N2	14 (20)
N3	7 (10)
N4	5 (7)

^a^n = 70

### DNA isolation

DNA for analysis was obtained from an archival pathological paraffin-embedded tumor and non-cancerous breast samples which were deparaffinized in xylene and rehydratated in ethanol and distilled water. The cancerous and non-cancerous breast tissue samples were fixed routinely in phormaldehyde, embedded in paraffin, cut into thin slices and stained with hematoxyline/eozine for pathological examination. In order to ensure, that the chosen histological material is representative for cancerous and non-cancerous tissue every tissue sample qualified for DNA extraction was initially checked by a pathologist. For tissue deparaffinization, 1,200 μl xylene was added to tissue section and agitated for 5 min, then centrifuged at 12,000 rpm for 10 min. The supernatant was removed and fresh xylene was added, and this step was repeated five times followed by washing with 100 % ethanol for 10 min and centrifuging at 12,000 rpm for 10 min. Then, the tissue pellet was air-dried. 180 µL of DNA extraction buffer solution ATL (Qiagen GmbH, Hilden, Germany) was added to the deparaffinized tissue in a 1.5 ml microcentrifuge tube followed by the DNA extraction step. Genomic DNA was prepared using QIAmp Kit (Qiagen GmbH, Hilden, Germany) according to the manufacturer instruction.

### Determination of hMSH2–Gly322Asp genotype

Polymorphism Gly322Asp (rs4987188) of the *hMSH2* gene was determined by PCR–RFLP, using primers: sense 5′-GTTTTCACTAATGAGCTTGC-3′, antisense 5′-GTGGTATAATCATGTGGGT-3′). The PCR was carried out in a PTC-100 TM (MJ Research, INC) thermal cycler. PCR amplification was performed in the final volume of 25 μl of reaction mixture, which contained 5 ng of genomic DNA, 0.2 μmol of each primer (ARK Scientific GmbH Biosystems, Darmstad, Germany), 2.5 mM of MgCl_2_, 1 mM of dNTPs and 1 unit of Taq Polymerase (Qiagen GmbH, Hilden, Germany). PCR cycle conditions were the following: 95 °C for 30 s, 60 °C for 30 s and 72 °C for 30 s, repeated in 30 cycles. PCR products were electrophoresed in a 2 % agarose gel and visualised by ethidium bromide staining. The cleavage with *Hin*fI produced fragments of 252 and 70/182 bp corresponding to the Gln and Asp alleles of the *hMSH2* gene, respectively.

### Determination of Asn127Ser genotype

Polymorphism Asn127Ser (rs17217772) of the *hMSH2* gene was determined by PCR–RFLP, using primers: two allele specific sense oligonucleotides 5′-TTAGGCTTCTCCTGGCAA-3′ for Asn variant and 5′-TTAGGCTTCTCCTGGCAG-3′ for Ser variant and antisense primer 5′-AGGAGAGCCTCAAGATTG-3′. The control PCR for each sample using sense primer 5′-AAAATTTTAAAGTATGTTCAAG-3′ and antisense primer described above was performed. The 210 and 264 bp control PCR products were electrophoresed in a 2 % agarose gel and visualised by ethidium bromide staining.

The PCR was carried out in a PTC-100 TM (MJ Research, INC) thermal cycler. PCR amplification was performed in the final volume of 25 μl of reaction mixture, which contained 5 ng of genomic DNA, 0.2 μmol of each primer (ARK Scientific GmbH Biosystems, Darmstad, Germany), 2.5 mM of MgCl_2_, 1 mM of dNTPs and 1 unit of Taq Polymerase (Qiagen GmbH, Hilden, Germany). PCR cycle conditions were the following: 95 °C for 30 s, 60 °C for 30 s and 72 °C for 40 s, repeated in 30 cycles.

### Statistical analysis

Logistic regression analysis was used to compute odds ratio (OR) and associated 95 % confidence interval (95 % CI) relating each of the single nucleotide polymorphism (SNP) as well as combinations of SNPs and another analysed factors presented in Table 1 to the risk of TNBC. The Hardy–Weinberg equilibrium (HWE; *p*2 + 2*pq* + *q*2 = 1), where *p* is the frequency of the variant allele (*q* = 1 − *p*) was tested by a goodness-of-fit Chi square test to compare the observed genotype frequencies with expected genotype frequencies in cancer-free controls. All multivariate models were adjusted for age, family history, menarche, parity, menopausal status, and use of contraceptive and menopausal hormones. *P* values <0.05 were considered significant. The disequilibrium coefficient *D*′ and *r* ^2^ values for all pair-wise combinations of SNPs were obtained using www.oege.org software. All the statistical analyses were performed, using the STATISTICA 6.0 software (Statsoft, Tulsa, Oklahoma, USA).

## Results

Table 3 shows genotype distribution values of *hMSH2* Gly322Asp polymorphisms in TNBC patients and controls. An association was found between the Asp/Asp and Gly/Asp genotypes of the Gly322Asp polymorphism of *hMSH2* gene and TNBC occurrence. Variant Asp allele of *hMSH2* decreased cancer risk.

**Table 3:** Distribution of genotypes and odds ratios (OR) of the Gly322Asp and Asn127Ser polymorphisms of the *hMSH2* gene in patients with TNBC and controls

Genotype	Patients (N = 70) N (%)	Controls (N = 70) N (%)	OR (95 % CI)^a^
*Gly322Asp*			
Gly/Gly	61 (87)	25 (36)	1.00 ref.
Gly/Asp	4 (6)	20 (28)	**0.08 (0.02–0.26)**
Asp/Asp	5 (7)	25 (36)	**0.08 (0.02–0.23)**
Gly	126 (90)	70 (50)	1.00 ref.
Asp	14 (10)	70 (50)	**0.11 (0.05–0.21)**
*Asn127Ser*			
Asn/Asn	20 (29)	18 (26)	1.00 ref.
Asn/Ser	33 (47)	33 (47)	0.90 (0.40–2.00)
Ser/Ser	17 (24)	19 (27)	0.80 (0.32–2.00)
Asn	73 (52)	69 (49)	1.00 ref.
Ser	67 (48)	71 (51)	0.89 (0.55–1.42)

Data in bold font are statistically significant

^a^Adjusted for age, family history, menarche, parity, menopausal status, and use of contraceptive and menopausal hormones

There were no differences in the genotype distributions between TNBC patients and controls for the *hMSH2* Asn127Ser polymorphism (see Table 3).

Among the controls, all genotype distributions did not differ significantly (*P* > 0.05) from those expected by the HWE. The observed genotype frequencies of *hMSH2* Asn127Ser SNP in the patients subjects were in agreement with HWE (*P* > 0.05; *χ* ^2^ = 0.217), but the observed genotype frequencies of *hMSH2* Gly322Asp SNP were not in agreement with HWE (*P* < 0.001; *χ* ^2^ = 22,491). It is caused by the very low abundance of the *hMSH2* Asp/Asp genotype in the examined Polish population.

Linkage disequilibrium (LD) analysis showed that the two SNPs were in LD (*D*′ = 1.0, *r* ^2^ = 0.1358). So, we have analyzed combined genotype of all polymorphism pairs. Table 4 shows the haplotypes distribution of *hMSH2*. The haplotypes analysis according to wild-type of Gly322Gly–Asn127Asn showed high frequency Gly322Gly–Asn127Ser, genotype. The combined Gly322Gly–Asn127Ser genotype increased the risk of TNBC (OR = 3.71, *P* = 0.017). Moreover, the combined Gly322Asp–Asn127Ser and Asp322Asp–Ser127Ser genotype decreased the risk of breast cancer occurrence.

**Table 4:** Haplotypes distribution and frequencies of *hMSH2* gene polymorphisms in TNBC patients and the controls

Haplotypes *hMSH2*-322–127	Patients (N = 70) N (%)	Controls (N = 70) N (%)	OR (95 % CI)^a^
Gly/Gly–Asn/Asn	20 (29)	18 (26)	1.00 ref.
Gly/Gly–Asn/Ser	33 (47)	8 (11)	**3.71 (1.36–10.10)**
Gly/Gly–Ser/Ser	8 (11)	NE	NE
Gly/Asp–Asn/Asn	NE	NE	NE
Gly/Asp–Asn/Ser	4 (6)	20 (29)	**0.18 (0.05–0.62)**
Gly/Asp–Ser/Ser	NE	NE	NE
Asp/Asp–Asn/Asn	NE	NE	NE
Asp/Asp–Asn/Ser	NE	5 (7)	NE
Asp/Asp–Ser/Ser	5 (7)	19 (27)	**0.24 (0.07–0.76)**

Data in bold font are statistically significant

*NE* not estimated

^a^Adjusted for age, family history, menarche, parity, menopausal status, and use of contraceptive and menopausal hormones

Histological grading was related to *hMSH2* polymorphisms. Histological grades were evaluated in all the cases (n = 70); grade 1—20 cases, grade 2—45 cases and grade 3—5 cases. Grades 2 and 3 were accounted together for statistical analysis (see Table 5).

**Table 5:** Dependence of genotypes and frequencies of *hMSH2* gene polymorphism alleles on tumour grade in TNBC patients^a^

Grade^b^	Triple-negative breast cancer patients		
	I (n = 20)	II + III (n = 50)	OR (95 % CI)^c^
	Number (%)	Number (%)	
*Gly322Asp*			
Gly/Gly	15 (75)	46 (92)	1.00 ref
Gly/Asp	2 (10)	2 (4)	3.06 (0.39–23.70)
Asp/Asp	3 (15)	2 (4)	4.60 (0.70–30.19)
Gly	32 (80)	94 (94)	1.00 ref
Asp	8 (20)	8 (8)	**2.93 (1.01–8.47)**
*Asn127Ser*			
Asn/Asn	6 (30)	14 (28)	1.00 ref
Asn/Ser	8 (40)	25 (50)	0.74 (0.21–2.59)
Ser/Ser	6 (30)	11 (22)	1.27 (0.32–5.05)
Asn	20 (50)	53 (53)	1.00 ref
Ser	20 (50)	47 (47)	1.13 (0.54–2.34)

Data in bold font are statistically significant

^a^n = 70

^b^According to Scarf–Bloom–Richardson criteria

^c^Adjusted for age, family history, menarche, parity, menopausal status, and use of contraceptive and menopausal hormones

No differences were observed in those groups, regarding either *hMSH2* Asn127Ser genotype or allele distributions. Some correlation was observed between the genotypes of *hMSH2*–Gly322Asp polymorphisms and breast cancer invasiveness. A statistically significant increase was observed, regarding Asp allele frequency (OR 2.93; 95 % CI 1.01–8.47, *P* = 0.043) in stage I patients, according to Scarf–Bloom–Richardson classification.

Table 6 shows the distribution of genotypes and the frequency of alleles in patients with different tumor size. A tendency for decreased risk of TNBC was observed with the occurrence of Asp allele of *hMSH2 p*olymorphism. That decrease was statistically significant (*P* < 0.05).

**Table 6:** *hMSH2* gene polymorphisms and TNBC progression^a^

	TNBC patients (n = 70)			TNBC patients (n = 70)		
	Tumor size			Node status		
	T1 + T2 N = 48	T3 + T4 N = 22	OR (95 % CI)^a^	N+ (n = 38)	N− (n = 32)	OR (95 % CI)^b^
	Number (%)	Number (%)		Number (%)	Number (%)	
*Gly322Asp*						
Gly/Gly	44 (92)	17 (77)	1.00 ref	31 (82)	30 (94)	1.00 ref
Gly/Asp	2 (4)	2 (9)	0.38 (0.05–2.96)	3 (8)	1 (3)	2.90 (0.28–29.4)
Asp/Asp	2 (4)	3 (14)	0.25 (0.03–1.67)	4 (10)	1 (3)	3.87 (0.40–36.6)
Gly	90 (94)	36 (83)	1.00 **r**ef	65 (86)	61 (95)	1.00 ref
Asp	6 (6)	12 (27)	**0.20 (0.07–0.57)**	11 (14)	3 (5)	3.41 (0.91–12.9)
*Asn127Ser*						
Asn/Asn	13 (27)	7 (32)	1.00 ref	12 (32)	8 (25)	1.00 ref
Asn/Ser	25 (52)	8 (36)	1.68 (0.49–5.67)	14 (36)	19 (59)	0.49 (0.15–1.52)
Ser/Ser	10 (21)	7 (32)	0.76 (0.20–2.91)	12 (32)	5 (16)	0.53 (0.16–1.72)
Asn	51 (53)	22 (50)	1.00 ref	20 (50)	35 (55)	1.00 ref
Ser	45 (47)	22 (50)	0.88 (0.43–1.80)	20 (50)	29 (45)	1.21 (0.54–2.66)

^a^T2 versus T3 + T4

^b^N− (node negative) versus N+ (node positive)

Moreover the distribution of genotypes and the frequency of alleles in patients with (N+) and without (N−) lymph node metastases are presented in Table 6. A tendency for an increased risk of TNBC was observed with the occurrence of Asp/Asp genotype and Asp allele of *hMSH2* polymorphism. That increase was, however, not statistically significant (*P* > 0.05) (see Table 6).

## Discussion

Breast cancer is the leading cause of tumour-related death among women worldwide [32]. Currently therapeutic approaches are limited by the development of drug resistance and progression of the majority of tumours to a more invasive and aggressive phenotype [33]. Resistance to anticancer agents that induce DNA damage has been associated with increased expression of DNA repair genes [34, 35] and the development of aggressive/metastatic behaviour in at least four different types of tumours [36–39].

Recently, using gene expression profiling of human primary malignant melanoma, Sarasin and Kauffman [37, 38] hypothesised that aberrant expression of genes connected with DNA repair pathways is associated with increased metastatic potential.

As mentioned in “Introduction” section MMR function leads to MSI, a type of genomic instability characterized by alterations in the length of microsatellite sequences distributed throughout the genome [40]. In fact, higher levels of DNA damage and deficient DNA repair may predispose individuals to cancer [41].

Commonly occurring SNPs in DNA repair genes have also been shown to incrementally contribute to cancer risk because of their critical role in maintaining genome integrity [42].

Many genetic changes in the form of SNPs have been identified in the MMR genes, but the function of these SNPs is largely unknown. Such alterations in MMR genes may have various effects on tumor phenotype, depending on where the alteration is located within the gene.

The aim of the present study was to evaluate associations between the risk of TNBC and polymorphisms in the *hMSH2* gene, encoding for key protein of MMR. In the present work we analysed two single nucleotide polymorphisms of the *hMSH2* DNA repair genes and tested the association between the distributions of their genotypes with TNBC.

In our study a weak association was found between the Asp/Asp (OR 0.08; 95 % CI 0.02–0.26) and Gly/Asp (OR 0.08; 95 % CI 0.02–0.23) genotype of the Gly322Asp polymorphism and TNBC occurrence.

Variant Asp allele of *hMSH2* decreased cancer risk. Moreover, the combined Gly322Asp–Asn127Ser and Asp322Asp–Ser127Ser genotype decreased the risk of breast cancer occurrence. Gly322Gly–Asn127Ser variant was associated with an elevated risk of TNBC in the Polish population. Additionally we observe correlation between studied Gly322Asp polymorphism and breast cancer progression evaluated by tumor size and Bloom–Richardson grading.

Our data suggest a protective role of the Asp allele of the Gly322Asp polymorphism of the *hMSH2* gene against the development of cancer and this function can be underlined by increasing the activity of MMR.

This result may suggest major contribution of the Gln322Asp polymorphism of the *hMSH2* gene in cancer development but more studies performed on larger population is needed to draw a final conclusion.

A positive correlation between polymorphism of the *hMSH2* gene and occurrence of cancer was reported in colorectal cancer [43–45], gastric cancer [46], lymphoma [47] and leukemia [48].

Poplawski et al. [49] suggest that 322Gly variant in the *hMSH2* gene may increase the risk of breast cancer.

However the role of *hMSH2* polymorphisms and breast cancer development is still unknown. To date, none studies have addressed the association between alterations in this region of the *hMSH2* gene and TNBC. Because a proper functioning of the *hMSH2* gene is important for the genomic stability, its alternations may be associated with higher cancer susceptibility.

In the recent studies, polymorphism of *hMSH2* may be associated with an elevated tumour risk in the Polish populations, regarding breast cancer [49], while there are still no data, which would be illustrating the significance of *hMSH2* polymorphism for TNBC development in other populations.

In the reported study, the Asn127Ser polymorphism of *hMSH2* gene was not correlated with triple-negative breast carcinoma progression. Our results are in line with the data from other reports [48]. *hMSH2* Asn127Ser gene variants were found to be not associated with breast cancer risk. This polymorphism was not related, either to tumor size or grade [36].

A limitation of the study was the relatively small population of participants. The sample for the present study comprised of 70 TNBC patients. This sample is only a very small proportion of the entire population of TNBC women in the country. Therefore the obtained results can not be considered as definitive and require further, more extensive evaluations, performed on bigger groups of patients. However, our preliminary results are fairly promising, indicating a significant role of the polymorphisms of *hMSH2* gene for TNBC development.

In conclusion in the present study, an association was identified between Gln322Asp polymorphism of *hMSH2* the incidence of TNBC. The obtained data suggest that the reported study may be the first observation of the polymorphisms in *hMSH2* genes, involved in the DNA repair pathway, to be associated with triple-negative breast carcinoma risk in the population of Polish women.

## References

[1] Vargo-Gogola T, Rosen JM (2007). Modelling breast cancer: one size does not fit all. Nat Rev Cancer.

[2] Veronesi U, Boyle P, Goldhirsch A (2005). Breast cancer. Lancet.

[3] Dapic V, Carvalho MA, Monteiro AN (2005). Breast cancer susceptibility and the DNA damage response. Cancer Control.

[4] Sørlie T, Perou CM, Tibshirani R (2001). Gene expression patterns of breast carcinomas distinguish tumor subclasses with clinical implications. Proc Natl Acad Sci USA.

[5] Sørlie T, Tibshirani R, Parker J (2003). Repeated observation of breast tumor subtypes in independent gene expression data sets. Proc Natl Acad Sci USA.

[6] Greenlee RT, Hill-Harmon MB, Murray T (2001). Cancer statistics. CA Cancer J Clin.

[7] Thomson TA, Hayes MM, Spinelli JJ (2001). HER-2/neu in breast cancer: interobserver variability and performance of immunohistochemistry with 4 antibodies compared with fluorescent in situ hybridisation. Mod Pathol.

[8] Gudmundsdottir K, Tryggvadottir L, Eyfjord JE (2001). GSTM1, GSTT1, and GSTP1 genotypes in relation to breast cancer risk and frequency of mutations in the p53 gene. Cancer Epidemiol Biomarkers Prev.

[9] Yee CJ, Roodi N, Verrier CS (1994). Microsatellite instability and loss of heterozygosity in breast cancer. Cancer Res.

[10] Shaw JA, Walsh T, Chappell SA (1996). Microsatellite instability in early sporadic breast cancer. Br J Cancer.

[11] Walsh T, Chappell SA, Shaw JA (1998). Microsatellite instability in ductal carcinoma in situ of the breast. J Pathol.

[12] Loeb LA, Loeb KR, Anderson JP (2003). Multiple mutations and cancer. Proc Natl Acad Sci USA.

[13] Loeb LA (2001). A mutator phenotype in cancer. Cancer Res.

[14] Loeb LA (1994). Microsatellite instability: marker of a mutator phenotype in cancer. Cancer Res.

[15] Hsieh P, Yamane K (2008). DNA mismatch repair: molecular mechanism, cancer, and ageing. Mech Ageing Dev.

[16] Skinner AM, Turker MS (2005) Oxidative mutagenesis, mismatch repair, and aging. Sci Aging Knowl Environ 9:re3 10.1126/sageke.2005.9.re3 15744047

[17] Karran P (1996). Microsatellite instability and DNA mismatch repair in human cancer. Semin Cancer Biol.

[18] Ben Yehuda A, Globerson A, Krichevsky S (2000). Ageing and the mismatch repair system. Mech Ageing Dev.

[19] Neri S, Gardini A, Facchini A (2005). Mismatch repair system and aging: microsatellite instability in peripheral blood cells from differently aged participants. J Gerontol A Biol Sci Med Sci.

[20] Krichevsky S, Pawelec G, Gural A (2004). Age related microsatellite instability in T cells from healthy individuals. Exp Gerontol.

[21] Esh Kuligina, Grigoriev M, Suspitsin E (2007). Microsatellite instability analysis of bilateral breast tumors suggests treatment-related origin of some contralateral malignancies. J Cancer Res Clin Oncol.

[22] Moinfar F, Beham A, Friedrich G (2008). Macro-environment of breast carcinoma: frequent genetic alterations in the normal appearing skins of patients with breast cancer. Mod Pathol.

[23] Spagnoletti I, Pizzi C, Galietta A (2004). Loss of hMSH2 expression in primary breast cancer with p53 alterations. Oncol Rep.

[24] Cai KQ, Albarracin C, Rosen D (2004). Microsatellite instability and alteration of the expression of hMLH1 and hMSH2 in ovarian clear cell carcinoma. Hum Pathol.

[25] Hishida A, Matsuo K, Hamajima N (2003). Polymorphism in the hMSH2 gene (gIVS 12–6T → C) and risk of non-Hodgkin lymphoma in a Japanese population. Cancer Genet Cytogenet.

[26] Oliveira C, Ferreira P, Nabais S (2004). E-cadherin (CDH1) and p53 rather than SMAD4 and Caspase-10 germline mutations contribute to genetic predisposition in Portuguesegastric cancer patients. Eur J Cancer.

[27] Bock N, Meden H, Regenbrecht M (2000). Expression of the mismatch repair protein hMSH2 carcinoma in situ and invasive cancer of the breast. Anticancer Res.

[28] Murata H, Khattar NH, Kang Y (2002). Genetic and epigenetic modification of mismatch repair genes hMSH2 and hMLH1 in sporadic breast cancer with microsatellite instability. Oncogene.

[29] Seitz S, Wassmuth P, Plaschke J (2003). Identification of microsatellite instability and mismatch repair gene mutations in breast cancer cell lines. Genes Chromosomes Cancer.

[30] Balogh GA, Russo IH, Russo J (2004). Truncation of the mismatch repair protein PMS2 during the neoplastic transformation of human breast epithelial cells in vitro. Int J Oncol.

[31] Balogh GA, Russo IH, Russo J (2003). Mutations in mismatch repair genes are involved in the neoplastic transformation of human breast epithelial cells. Int J Oncol.

[32] Jemal A, Siegel R, Ward E (2007). Cancer statistics, 2007. CA Cancer J Clin.

[33] Mimeault M, Batra SK (2010). New promising drug targets in cancer- and metastasis-initiating cells. Drug Discov Today.

[34] Liu C, Zhou S, Begum S (2007). Increased expression and activity of repair genes TDP1 and XPF in non-small cell lung cancer. Lung Cancer.

[35] Xu ZY, Loignon M, Han FY (2005). Xrcc3 induces cisplatin resistance by stimulation of Rad51-related recombinational repair, S-phase checkpoint activation, and reduced apoptosis. J Pharmacol Exp Ther.

[36] Ganguly A, Shields CL (2010). Differential gene expression profile of retinoblastoma compared to normal retina. Mol Vis.

[37] Kauffmann A, Rosselli F, Lazar V (2008). High expression of DNA repair pathways is associated with metastasis in melanoma patients. Oncogene.

[38] Sarasin A, Dessen P (2010). DNA repair pathways and human metastatic malignant melanoma. Curr Mol Med.

[39] Wang Y, Klijn JG, Zhang Y (2005). Gene-expression profiles to predict distant metastasis of lymph-node-negative primary breast cancer. Lancet.

[40] de la Chapelle A (2004). Genetic predisposition to colorectal cancer. Nat Rev Cancer.

[41] Moses RE (2001). DNA damage processing defects and disease. Annu Rev Genomics Hum Genet.

[42] Ford BN, Ruttan CC, Kyle VL (2000). Identification of single nucleotide polymorphisms in human DNA repair genes. Carcinogenesis.

[43] Starinsky S, Figer A, Ben-Asher E (2005). Genotype phenotype correlations in Israeli colorectal cancer patients. Int J Cancer.

[44] Hu F, Li D, Wang Y, Yao X (2013). Novel DNA variants and mutation frequencies of hMLH1 and hMSH2 genes in colorectal cancer in the Northeast China population. PLoS One.

[45] Zhou JN, Wang DQ, Song L (2010). Association of IVS10 + 12G > A polymorphism in hMSH2 gene with colorectal cancer in Chinese. Zhonghua Yi Xue Yi Chuan Xue Za Zhi.

[46] Sud R, Wells D, Talbot IC (2001). Genetic alterations in gastric cancers from British patients. Cancer Genet Cytogenet.

[47] Hishida A, Matsuo K, Hamajima N (2003). Polymorphism in the hMSH2 gene (gIVS 12–6T → C) and risk of non-Hodgkin lymphoma in a Japanese population. Cancer Genet Cytogenet.

[48] Worrillow LJ, Travis LB, Smith AG (2003). An intron splice acceptor polymorphism in hMSH2 and risk of leukemia after treatment with chemotherapeutic alkylating agents. Clin Cancer Res.

[49] Poplawski T, Zadrozny M, Kolacinska A (2005). Polymorphisms of the DNA mismatch repair gene HMSH2 in breast cancer occurence and progression. Breast Cancer Res Treat.

